# Sex, pregnancy and aortic disease in Marfan syndrome

**DOI:** 10.1371/journal.pone.0181166

**Published:** 2017-07-14

**Authors:** Marjolijn Renard, Laura Muiño-Mosquera, Elise C. Manalo, Sara Tufa, Eric J. Carlson, Douglas R. Keene, Julie De Backer, Lynn Y. Sakai

**Affiliations:** 1 Center for Medical Genetics Ghent, Ghent University, Ghent, Belgium; 2 Department of Molecular & Medical Genetics and Biochemistry & Molecular Biology, Shriners Hospital for Children, Portland, Oregon, United States of America; 3 Micro-Imaging Center, Shriners Hospital for Children, Portland, Oregon, United States of America; Max Delbruck Centrum fur Molekulare Medizin Berlin Buch, GERMANY

## Abstract

**Background:**

Sex-related differences as well as the adverse effect of pregnancy on aortic disease outcome are well-established phenomena in humans with Marfan syndrome (MFS). The underlying mechanisms of these observations are largely unknown.

**Objectives:**

In an initial (pilot) step we aimed to confirm the differences between male and female MFS patients as well as between females with and without previous pregnancy. We then sought to evaluate whether these findings are recapitulated in a pre-clinical model and performed in-depth cardiovascular phenotyping of mutant male and both nulliparous and multiparous female Marfan mice. The effect of 17β-estradiol on fibrillin-1 protein synthesis was compared *in vitro* using human aortic smooth muscle cells and fibroblasts.

**Results:**

Our small retrospective study of aortic dimensions in a cohort of 10 men and 20 women with MFS (10 pregnant and 10 non-pregnant) confirmed that aortic root growth was significantly increased in the pregnant group compared to the non-pregnant group (0.64mm/year vs. 0.12mm/year, p = 0.018). Male MFS patients had significantly larger aortic root diameters compared to the non-pregnant and pregnant females at baseline and follow-up (p = 0.002 and p = 0.007, respectively), but no significant increase in aortic root growth was observed compared to the females after follow-up (p = 0.559 and p = 0.352). In the GT-8/+ MFS mouse model, multiparous female Marfan mice showed increased aortic diameters when compared to nulliparous females. Aortic dilatation in multiparous females was comparable to Marfan male mice. Moreover, increased aortic diameters were associated with more severe fragmentation of the elastic lamellae. In addition, 17β-estradiol was found to promote fibrillin-1 production *by* human aortic smooth muscle cells.

**Conclusions:**

Pregnancy-related changes influence aortic disease severity in otherwise protected female MFS mice and patients. There may be a role for estrogen in the female sex protective effect.

## Introduction

Marfan syndrome (MFS, OMIM #154700) is a heritable connective tissue disorder caused by mutations in the fibrillin-1 (*FBN1*) gene (OMIM 134797) [1], which encodes a major component of extracellular microfibrils [2, 3]. MFS is a pleiotropic disorder affecting multiple organ systems, including the skeletal, ocular, and cardiovascular system. Cardiovascular manifestations in Marfan patients include thoracic aortic aneurysms and dissections (TAAD) most typically at the level of the sinuses of Valsalva, mitral valve prolapse and subclinical cardiomyopathy and arrhythmias [4–8]. Drivers of progressive aortic dilatation are largely unknown.

Previously, it was shown that sex impacts aortic disease in MFS. One study investigating 965 probands with pathogenic *FBN1* mutations (median age: 22 years (11–34), 53% males) found that men below the age of 30 were at higher risk for aortic dilatation and aortic events (aortic dissection or the need for prophylactic surgery) compared to women [9]. Further evidence for male sex as a risk factor in humans with MFS was provided by data from the Dutch Concor study, showing an increased incidence of aortic surgery at baseline (38.0 versus 19.4%) and during follow-up (24.0 versus 15.1%) in men compared to women [10]. In surgical series, on the other hand, male sex was not associated with a risk for recurrent interventions [11, 12]. However, it is not clear whether MFS women are protected against aortic disease or MFS men are more prone to aortic disease.

Several retrospective studies in MFS patients indicated that pregnancy is considered as a risk factor for developing aortic dissection [13–18]. A recent retrospective study by Roman *et al*., using data from the GenTAC registry, reported an 8-fold increased risk of type A or B aortic dissection in pregnant MFS women compared to never-pregnant MFS women [19]. This risk was especially increased during the immediate postpartum period. Of note, many of the women experiencing pregnancy-related aortic dissection had not been diagnosed with MFS prior to the event.

Until now, few prospective studies have been conducted [20–23]. These studies reported a low incidence of aortic complications during pregnancy in women with a prior diagnosis of MFS, an initial diameter of the aortic root ≤ 45 mm, and appropriate follow-up during pregnancy. During a total of 133 pregnancies, only 4 aortic dissections and 2 carotid artery dissections were reported [20–23]. Donnelly and colleagues noted that in the long-term, pregnancy does increase the risk of aortic complications in MFS women [20]. In addition, this group and others also reported increased aortic growth rate during pregnancy [20, 21].

The influence of sex and pregnancy on cardiovascular disease in MFS is becoming increasingly studied. Preclinical animal research has, however, not followed suit. To date, no reports have been published on the influence of sex or pregnancy on aortic disease in MFS mice. Both *in vivo* and *in vitro* data obtained using mouse models could provide important insights into disease mechanisms, which could lead to new options for clinical management and treatment of patients.

To date, several *Fbn1* mutant mouse models have been reported. These range in clinical severity from the absence of any phenotypic features of MFS (*Fbn1*^*mgN/+*^ and *Fbn1*^*H1Δ/H1Δ*^) to mild MFS (*Fbn1*^*C1039G/+*^) and premature death due to aortic rupture (*Fbn1*^*mgN/mgN*^, *Fbn1*^*mgΔ/mgΔ*^, *Fbn1*^*mgR/mgR*,^ *Fbn1*^*GT-8/GT-8*^) [24–29]. Here, we studied the *Fbn1*^*GT-8/+*^ (GT-8/+) mouse model. The mutant allele in this mouse model is truncated by the addition of an enhanced Green Fluorescent Protein tag. Homozygous GT-8/GT-8 mice die during the early postnatal period, similar to the complete null mouse (mgN/mgN) [24]. In heterozygosity, GT-8/+ mice have a normal life span and present progressive fragmentation of the elastic lamellae of the aorta as early as 2 months of age and fragmentation of microfibrils in skin, skeletal muscle and tendon [25]. Gross *ex vivo* examination of the aorta indicated the presence of aortic aneurysms in this mouse model. However, no systematic analyses of the aortic aneurysms were performed.

In this study, the influence of sex and of pregnancy was investigated in MFS humans and mice. Our results confirmed larger baseline aortic diameters in MFS men compared to MFS women and increased aortic growth in pregnant MFS women compared to non-pregnant MFS women. Similarly, male MFS mice have more severe aortic disease than female mice and pregnancy adversely affects aortic disease. In addition, the female hormone 17β-estradiol stimulates aortic smooth muscle cells to produce fibrillin-1 *in vitro*, providing a hypothesis for a female protective effect against aortic disease due to increased production of fibrillin-1.

## Material and methods

### Clinical studies

Aortic root dimensions were retrospectively recorded from a cohort of 20 women and 10 men with MFS. Dimensions were measured with transthoracic echocardiography at the level of the sinuses of Valsalva according to the guidelines (leading-to-leading edge, end-diastole). Aortic root diameters were determined before pregnancy for the pregnant group or at an equivalent time point for the non-pregnant and male group. These measurements are referred to as the baseline measurements. In addition, measurements of the last echocardiography at follow-up were used (mean follow-up time of 5,6 years (1,4–9,8 years)). Progression of aortic root dilation was compared between 10 pregnant women (total of 13 pregnancies), 10 non-pregnant women, and 10 men. Non-pregnant women and men were selected from our population of MFS patients and were matched to the pregnant group for age.

The retrospective study in MFS patients was approved by the local ethical committee of the Ghent University Hospital (permit number 2015–0411), and the procedures followed were in accordance with institutional guidelines and conform to the principles outlined in the Declaration of Helsinki. Participants included in the study gave written informed consent prior to inclusion.

### Mice

Details of the generation of GT-8/+ mice were described previously [25]. Male and female GT-8/+ mice on a pure C57BL/6 background were used for this study. Polymerase chain reaction-based genotyping was performed as previously described [25].

All experiments on mice were carried out in strict accordance with the recommendations in the Guide for the Care and Use of Laboratory Animals of the National Institutes of Health. The protocols were approved by the Oregon Health & Science Institutional Animal Care and Use Committee (Permit Number: ISO1405). All ultrasound analyses were performed under isoflurane anesthesia, and all efforts were made to minimize suffering. Mice were sacrificed by means of CO_2_ overdose (1.0L/min) and cervical dislocation.

### Cardiovascular ultrasound

Ultrasound imaging of the aorta of male and female wild-type and heterozygous GT-8/+ mice was performed at the age of 8 and 12 months. Females were either nulliparous or allowed to breed; number of pregnancies ranged from 1–4, average litter size was 6.4 pups). Number of animals included at 8 months of age: n = 8 wild-type males; n = 11 GT-8/+ males; n = 13 nulliparous wild-type females; n = 5 multiparous wild-type females; n = 8 nulliparous GT-8/+ females; n = 19 multiparous GT-8/+ females. Number of animals included at 12 months of age: n = 10 wild-type males; n = 12 GT-8/+ males; n = 13 nulliparous wild-type females; n = 2 multiparous wild-type females; n = 8 nulliparous GT-8/+ females: n = 14 multiparous GT-8/+ females. Ultrasound studies were performed under general anaesthesia (1–1.5% isoflurane mixed with 0.5L/min 100% O_2_) using a dedicated ultrasound apparatus (Vevo 2100, Visualsonics) equipped with a high-frequency linear array transducer (MS 550D, frequency 22-55MHz). Aortic dimensions were measured at the level of the sinuses of Valsalva, proximal and distal ascending aorta, transverse arch and descending thoracic aorta. Ultrasound images were analysed by one individual blinded to the genotype.

### Light microscopy

Wild-type and GT-8/+ male and female mice were sacrificed at the age of 8 months (n = 7 M wild-type; n = 6 M GT-8/+; n = 8 nulliparous F wild-type; n = 5 nulliparous F GT-8/+; n = 4 multiparous F wild-type; and n = 6 multiparous F GT-8/+), and between 12 and 16 months (n = 3 M wild-type; n = 7 M GT-8/+; n = 7 nulliparous F wild-type; n = 11 nulliparous F GT-8/+; n = 3 multiparous F wild-type; and n = 9 multiparous F GT-8/+) by means of CO_2_ overdose (1.0L/min) and cervical dislocation. In situ aortae were flushed with phosphate buffered saline (PBS) and dissected (including the aortic root up to the proximal part of the descending thoracic aorta). Aortas were fixed for a minimum of 12 hours at 4°C in 1.5% glutaraldehyde/1.5% formaldehyde with 0.05% tannic acid then rinsed in Dulbecco’s serum free media (41966–029, Gibco^™^). Subsequently, the aortae were post-fixed for 24 hours in media buffered 1% osmium tetroxide. Assisted by a Pelco Biowave Pro microwave processor, the tissues were dehydrated in a graded series of ethanol (30%-100% at 100W for 1min/step), rinsed in propylene oxide (2x at 100W for 1min/step), infiltrated in 1:1 and 1:3 propylene oxide:Spurr’s epoxy (250W for 15min/step) then in 3 changes of 100% Spurr’s epoxy (250W for 15min/step followed by overnight at 4°C) before embedding in fresh Spurr’s epoxy. 0.5 μm thick sections of the aortic root were stained with toluidine blue and basic fuchsin and photographed on a Leica DMIRE2 inverted microscope fitted with a 10x objective, NA = 0.3. Images were collected using a QImaging Micropublisher camera. The micrographs were analyzed to count fragmentation and major breaks. Fragmentation was measured by counting the number of breaks in all lamellae in full cross-sections of aortae. Breaks larger than what could be a plane-of-section artifact were counted. Major breaks were defined as breaks that spanned more than 3 layers of elastic lamellae. The number of fragments and large breaks per whole aorta section were counted by 2 separate individuals blinded to the genotype and averaged.

### *In vitro* hormone assay and ELISA

Commercially available human aortic smooth muscle (CC-2571, Lonza) and fibroblast cells (CRL 1537, American Type Culture Collection) were cultured in 24-well plates overnight. Subsequently, the cells were treated with 1, 2, 4 or 8 nmol/L estrogen (17-β estradiol from Sigma, E8875) or 100 ng/ml TGFβ-1 (240-B, R&D Systems) for 24 hours. Untreated cell cultures were used as a control. Medium was collected and a modified sandwich ELISA was used to quantitate secreted fibrillin-1 in cell culture medium as described previously [30]. Two antibody pairs consisting of a biotinylated (B) capture antibody and an alkaline phosphatase (AP)-conjugated detector antibody that recognize fibrillin-1 were used in this modified sandwich ELISA, B15-AP201 and B15-AP78. B15 recognizes the proline-rich region; AP201 recognizes the sixth calcium binding epidermal growth factor domain; and AP78 recognizes the second calcium binding epidermal growth factor domain of fibrillin-1. Custom fibrillin-1 antibodies have been described previously [31, 32]. In a first step, 96-well plates were coated for 18 hours at 4°C with 0.3 μg/mL Pierce^™^ streptavidin (21135, ThermoFisher Scientific) in 0.05M Na2CO3, 0.05M NaHCO3, pH 9.6. Subsequently, the plates were incubated for 1 hour at 25°C with 0.25 μg/ml biotinylated monoclonal antibody B15 in 50mM Tris, 150 mM NaCl, pH 7.4, 0.025% Tween20 (TBST) supplemented with 5% fetal bovine serum (FBS), followed by 20 hours incubation at 4°C with untreated or treated culture media samples. Washes with TBST were performed between each step to remove unbound material. All samples were run in duplicate. Alkaline phosphatase-conjugated detector antibodies (AP201 and AP78 at 0.05 μg/ml in TBST with 5% FBS) were used to complete the sandwich (1 hour incubation at 25°C). A standard curve was developed using a serial dilution of recombinant fibrillin-1 peptide rF11 (N-terminal half of fibrillin-1) in TBST with 10% FBS. To develop a colorimetric signal, 50 μl of Invitrogen ELISA Amplification System (19589–019) substrate was added to each well for 15 minutes, followed by 50 μl of Invitrogen ELISA Amplification System amplifier for 7 minutes. The reaction was stopped by adding 50 μl of H_2_SO_4_. The colorimetric signal was read on an Emax plate reader (Molecular Devices) at 490nm. The average absorbance of each sample duplicate was computed and converted to μg/mL with an equation generated from the standard curve. A 4-parameter log logistic curve was fit to the standards. Graphs were generated using GraphPad Prism software 5.04.

### Statistics

Normal distribution of the human and mice ultrasound measurements was assessed by calculating the mean, median, skewness and kurtosis per dataset. For normal distributed ultrasound values, as well as histological and ELISA data, one-way ANOVA followed by a post-hoc Bonferroni, Tukey or Dunnett’s test was performed. For ultrasound values that were not normally distributed, the Mann-Whitney-U or the Kruskal-Wallis test was applied. For nominal variables the Chi-square test was used. Results are shown as mean ± standard deviation, unless the values were not normally distributed in which case median ± interquartile range are shown. All values in mice are absolute measures, not corrected for length or weight. A p-value of <0.05 was used to define statistical significance (two-sided). GraphPad Prism version 5.04 and 5.02 was used for the statistical analysis of the ultrasound data in mice, and the histological and ELISA analyses, respectively. The SPSS version 23 was used for the statistical analysis of the human ultrasound data. All graphs were generated with the GraphPad Prism version 5.04 or 5.02.

## Results

### Aortic root diameters in female and male Marfan patients

**Fig 1:**
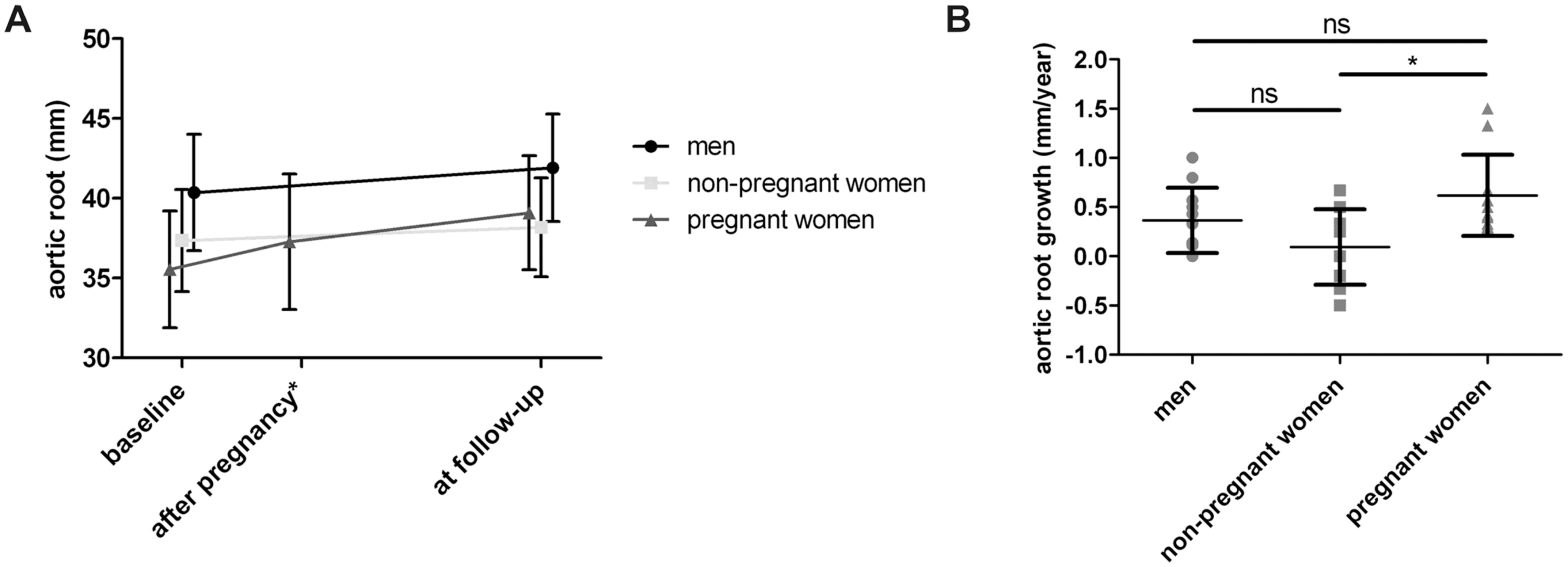
Aortic root characteristics in MFS patients. A. At baseline, aortic root diameters are higher in men compared to women. Non-pregnant women have higher aortic root diameters at baseline compared to pregnant women. After several years of follow-up, aortic root diameters increase in men and pregnant women, but these barely increase in non-pregnant women. Time interval between baseline and after pregnancy is 1.8 months, and between after pregnancy and follow-up is 5.5 years. * Values were only available for the pregnant women group at this time point. B. The growth rate of the aortic root, measured at baseline and last follow-up, was significantly higher in the pregnant group in comparison to the non-pregnant group. No significant difference in aortic growth rate was observed between the men and women. Results are shown as means ± standard deviation, unless the values were not normally distributed in which case median ± interquartile range are shown. ns: not significant; * p-value <0.05.

We first tested previously published findings of the influence of sex and pregnancy in MFS patients using our Ghent cohort. We conducted a retrospective study of aortic dimensions in an age-matched sample of 20 women with MFS (10 out of 20 women had been pregnant with a total of 13 pregnancies) and 10 MFS men (Table 1). At baseline, aortic root diameters were significantly higher in the men compared to the non-pregnant and pregnant women (p = 0.040 and p = 0.002, respectively) (Fig 1A). Aortic diameters were higher in the non-pregnant group compared to the pregnant group, although this difference was not significant (mean 36.9 mm versus 35.0 mm; p = 0.623) (Fig 1A). This finding possibly reflects the influence of aortic diameter in the decision to carry a pregnancy. No aortic dissection was observed during pregnancy. Several years of follow-up (mean 5.6 years) revealed that the growth rate of the aortic root was significantly higher in the pregnant group in comparison to the non-pregnant group (mean 0.64 mm/year versus 0.12 mm/year, p = 0.018) (Fig 1B). No significant difference in aortic growth rate was observed between the men and non-pregnant (p = 0.559) and pregnant (p = 0.352) women. Maximal aortic diameter progression was observed in the pregnant group during the first period after pregnancy and stabilized thereafter (Fig 1A). Healthy pregnant and non-pregnant women and men were not included in this study. Data on the aorta during pregnancy in non-MFS women have been published elsewhere [33].

**Table 1:** Characteristics of the aortic root of MFS patients at baseline and after several years of follow-up.

	men	women		p-value
		non-pregnant	pregnant	
n	10	10	10	
age (year)	26 (±10)	27 (±6)	27 (±3)	0.899
aortic root at baseline (mm)	40.8 (±3.52)	36.9 (±2.97)	35 (±3.40)	**0.002**^a^
Z-scores at baseline	3.33 (±1.41)	3.19 (±1.65)	1.32 (±0.76)	**0.026**
aortic root at follow-up (mm)	42.5 (±2.80)	37.9 (±3.10)	38.7 (±3.52)	**0.007**^b^
follow-up time (year)	5.00 (±2.75)	5.09 (±2.66)	6.63 (±3.74)	0.341
aortic root growth rate (mm/year)	0.36 (±0.35)	0.2 (±0.39)	0.64 (±0.42)	**0.022**^c^

Results are given as means with the standard deviation between brackets. Significant p-values (<0.05) are indicated in bold.

^a^ Values are significantly different between men and non-pregnant and pregnant women (p = 0.040 and p = 0.002, respectively), but not between the non-pregnant and pregnant women (p = 0.623).

^b^ Values are also significantly different when pairwise comparing men and non-pregnant and pregnant women (p = 0.010 and p = 0.038, respectively).

^c^ Significant difference only between non-pregnant and pregnant women (p = 0.018). No significant difference was observed between men and non-pregnant (p = 0.559) or pregnant women (p = 0.352).

### Aortic dimensions in male and female GT-8/+ mice

**Fig 2:**
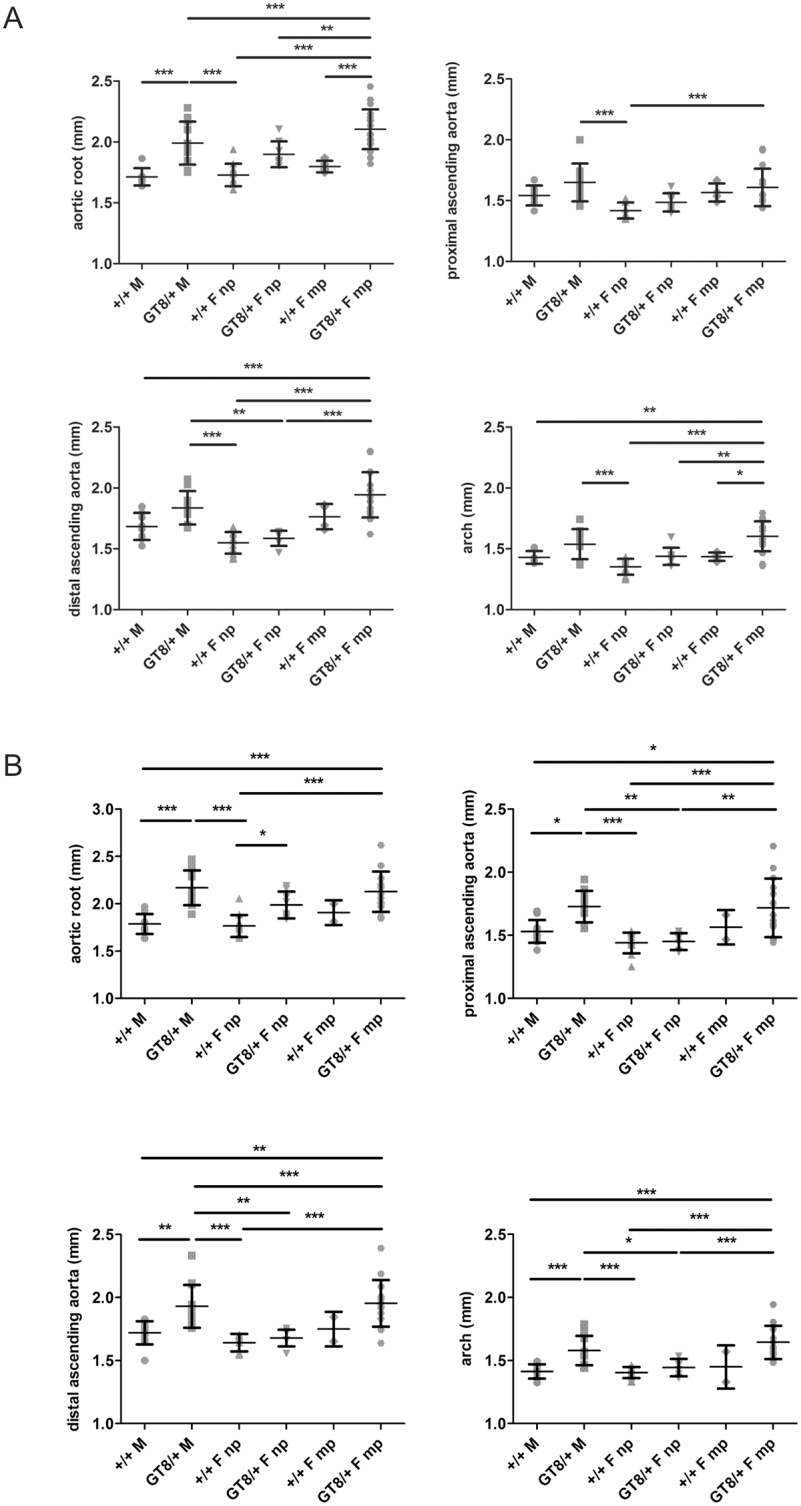
Aortic dimensions of wild-type (+/+) and heterozygous GT-8/+ males (M) and nulliparous (np) and multiparous (mp) females (F) at age 8 (panel A) and 12 months (panel B). Male and multiparous female GT-8/+ mice show more severe aortic disease than wild-type mice and nulliparous GT-8/+ females at both time points. Nulliparous GT-8/+ females differed only significantly from the nulliparous wild-type females at the level of the aortic root at 12 months. In addition, nulliparous females have smaller aortic diameters than the GT-8/+ males. Black lines represent the mean ± standard deviation. Non-significant differences are not indicated in the figure. p-values: * <0.05, ** <0.005, *** <0.001, **** <0.0001.

Ultrasound analyses of male heterozygous GT-8/+ mice at the age of 8 and 12 months demonstrated larger aortic diameters at all levels compared to age-matched male wild-type littermates (p<0.05 at the level of the aortic root at 8 months; and the aortic root, proximal and distal ascending aorta and arch at 12 months) (Fig 2 and S1 Table). In fact, a trend towards larger aortic diameters in male GT8/+ mice was observed as early as 1 month of age, with aortic diameters significantly increased compared to wild-type male mice at later time points (S2 Table). Increased aortic root, ascending and aortic arch diameters were also observed in 8 and 12 months old multiparous GT-8/+ females compared to nulliparous GT-8/+ females (p<0.05 at the level of the aortic root, distal ascending aorta and arch at 8 months; and proximal and distal ascending aorta and arch at 12 months) (Fig 2 and S1 Table). Nulliparous GT-8/+ females differed only significantly from the wild-type nulliparous females at the level of the aortic root (age 12 months). Aortic disease severity is similar between GT-8/+ males and GT-8/+ multiparous females. However, aortic diameters at the level of the aortic root and distal ascending aorta (8 months) and distal ascending aorta and arch (12 months) are significantly higher in multiparous GT-8/+ females compared to GT-8/+ males (Fig 2 and S1 Table). In contrast, nulliparous GT-8/+ females are less severely affected than GT-8/+ males at both time points (p<0.05 at the level of the distal ascending aorta at 8 months, and ascending and arch at 12 months). Mean aortic diameters are slightly higher in multiparous compared to nulliparous wild-type females, however, these differences are not significant at 8 months of age (Fig 2 and S1 Table). Due to the low n no statistics could be done for the 12 months time point. The number of pregnancies (range 1–4) and the time between the last pregnancy and the ultrasound analysis (range 0.5–6 months) did not have an effect on aortic disease severity.

The natural history of this cohort of GT-8/+ heterozygous mice showed that none of the mice died from aortic dissection/rupture. Two male GT-8/+ mice presented mild aortic valve regurgitation associated with aortic dilatation (at 8 and 12 months of age respectively). Three GT-8/+ multiparous female mice developed mild aortic valve regurgitation (1 at 8 months and 2 at 12 months of age) and two had significant aortic valve regurgitation with holodiastolic flow reversal in the descending aorta at the age of 8 months. The mitral valve was not affected in either of these two groups. None of the GT-8/+ nulliparous females or wild-type mice (both males and females) developed valvular problems.

### Sex- and pregnancy-related changes in the aortic wall of GT-8/+ mice

**Fig 3:**
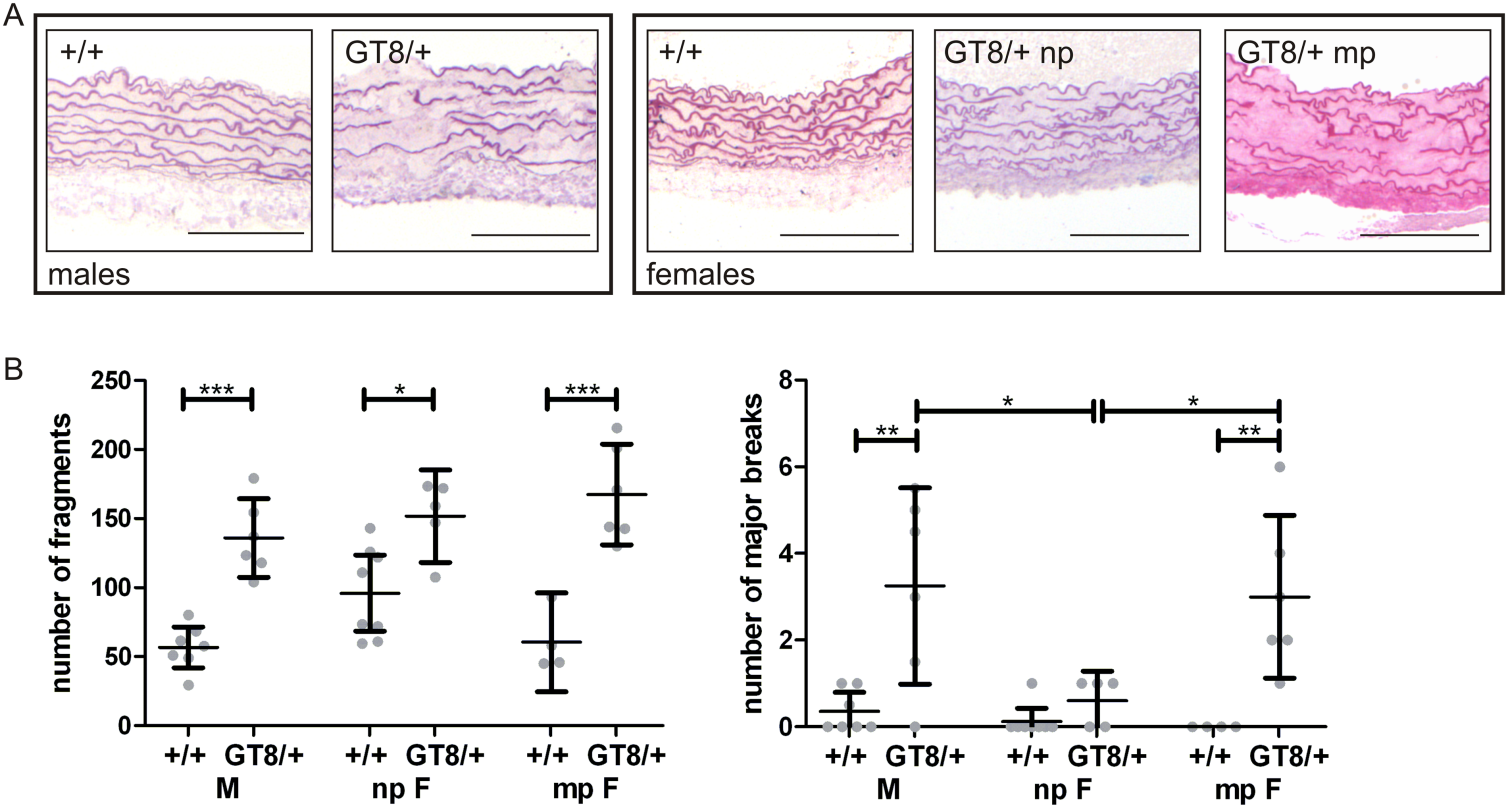
Aortic root morphology in wild-type and heterozygous GT-8/+ mice at the age of 8 months. A. The aortic elastic lamellae of GT-8/+ mice show clear fragmentation compared to the wild-type controls. Major breaks spanning at least 3 consecutive layers of elastic lamellae can be found in male and multiparous female GT-8/+ mice. Scale bars = 100 μm. B. At the age of 8 months, GT-8/+ male mice show significantly more fragmentation of the aortic elastic lamellae (left graph) and an increased number of major breaks (right graph) compared to wild-type littermates. Similarly, nulliparous and multiparous female GT-8/+ mice both show significantly more fragmentation of the aortic elastic lamellae compared to wild-type (left graph). The number of major breaks in the aortic root of multiparous GT-8/+ female mice is, however, significantly higher than in nulliparous GT-8/+ female mice and multiparous wild-type female mice (right graph). Each symbol represents a single animal. Means with 95% confidence intervals are shown. F: female; M: male; np: nulliparous; mp: multiparous; p-values: * <0.05, ** <0.005, *** <0.001.

Heterozygous GT-8/+ mice showed progressive fragmentation of the elastic lamellae of the aortic root, beginning at two months of age, increasing with age [25]. In the present study, fragmentation of the elastic lamellae was quantitated. 8 months old male GT-8/+ mice showed many more small breaks in the elastic lamellae compared to wild-type males (Fig 3). Similarly, 8 month old nulliparous and multiparous female GT-8/+ mice showed more severe fragmentation of the elastic lamellae of the aortic root, compared to wild-type females (Fig 3). No significant difference in the numbers of small breaks (around 150 per cross-section) was seen between male GT-8/+ mice and nulliparous or multiparous female GT-8/+ mice at this age (Fig 3).

By 8 months of age, the aorta of GT-8/+ mice also showed the presence of major breaks, defined as breaks spanning at least three consecutive layers of elastic lamellae. A marked difference was seen between nulliparous and multiparous GT-8/+ females when comparing the number of major breaks in the aortic root. Multiparous GT-8/+ females had significantly more major breaks in their aortic elastic lamellae compared to the nulliparous females. The number of major breaks in multiparous GT-8/+ females was comparable to GT-8/+ males at the age of 8 months (Fig 3). Similarly, older GT-8/+ mutant mice (ranging from 12–16 months) presented with more major breaks than the wild-type controls (ANOVA p = 0.0002) (S2 Fig). Multiparous GT-8/+ females presented more major breaks than nulliparous GT-8/+ females and GT-8/+ males, with only the latter comparison being significantly different. Compared to the 8 months old time point, male GT-8/+ mice show a similar number of major breaks, but both nulliparous and multiparous female GT-8/+ mice are characterized by double the amount of major breaks. Nulliparous GT-8/+ females thus catch up to the GT-8/+ males at advanced age (S2 Fig). This increase in aortic root major breaks in nulliparous GT-8/+ females is consistent with the significant increase in aortic root size found in 12 month old nulliparous mutant females compared to wild-type (Fig 2).

### Estrogen promotes fibrillin-1 production *in vitro*

**Fig 4:**
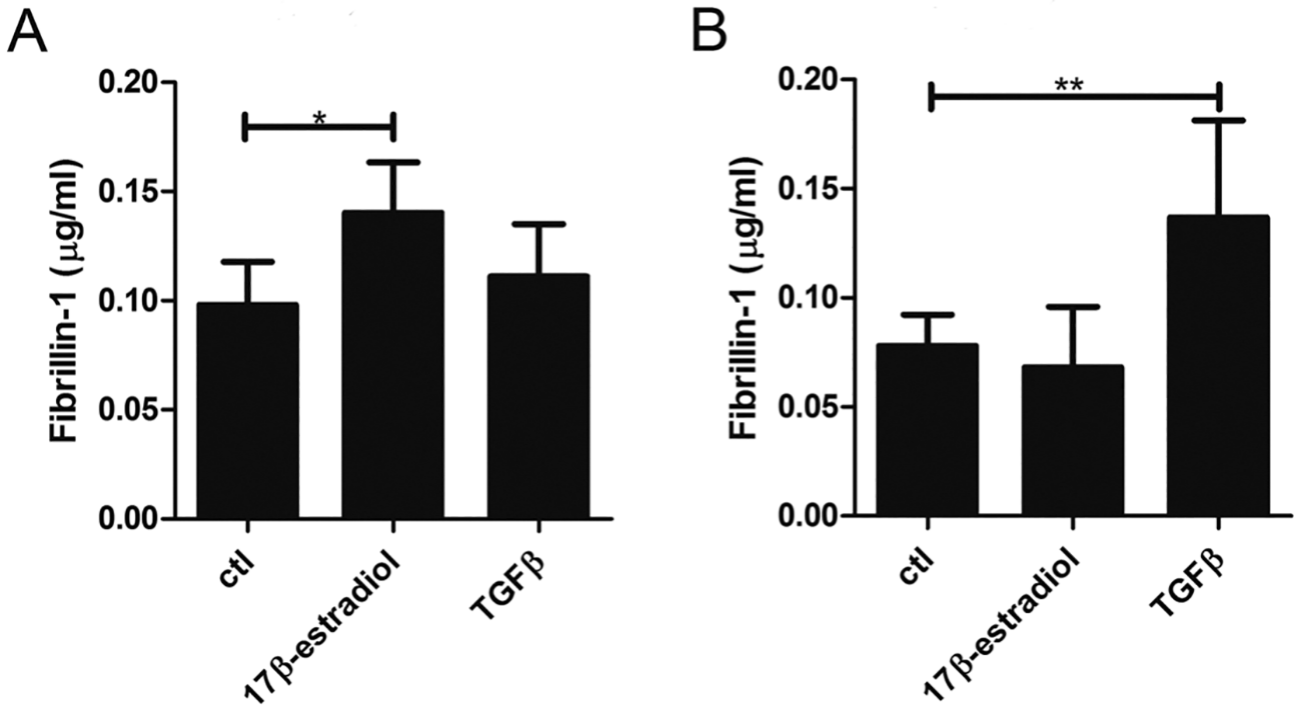
Fibrillin-1 production by (A) human aortic smooth muscle cells (HASMC) and (B) fibroblasts. Cells were treated with 17β- estradiol and TGFβ-1 and compared to untreated control cells. Higher concentrations of 17β-estradiol (4–8 nmol/L) compared to control (ctl) (0, 1 or 2 nmol/L 17β-estradiol) promoted fibrillin-1 production by HASMC. In contrast, fibrillin-1 production by fibroblasts was stimulated by TGFβ-1 but not by 17β-estradiol. Results shown were obtained using the antibody pair B15-AP201. Error bars (standard deviation) are based on combining two separate experiments with HASMC and three separate experiments with fibroblasts. In each experiment, samples were tested in duplicate. Significant p-values are marked with an *.

Since women and female mice with MFS seem to have milder aortic disease than men and male mice, we tested whether 17β-estradiol may be in part accountable for this protective effect by influencing fibrillin-1 production. Fibrillin-1 production was significantly increased upon administration of 17β-estradiol (4 or 8 nmol/L) to the medium of human aortic smooth muscle cells (HASMC) compared to untreated HASMC or HASMC treated with low concentrations of 17β-estradiol (1 or 2 nmol/L) (Fig 4A). In comparison, Transforming Growth Factor-β (TGFβ)-1, known to promote fibrillin-1 fibril formation by fibroblasts, did not induce HASMC to increase fibrillin-1 production. As a control, fibroblasts were also treated with 17β-estradiol and TGFβ-1 (Fig 4B). While TGFβ-1 increased fibrillin-1 synthesis and secretion by fibroblasts, 17β-estradiol did not. The magnitude of TGFβ-1 induction of fibrillin-1 production by fibroblasts was similar to the magnitude of fibrillin-1 production induced by 17β-estradiol in HASMC.

## Discussion

To date, a limited number of clinical studies have reported on the impact of sex on aortic disease in MFS and related Heritable Thoracic Aortic Disease. It has, for example, been shown that MFS men are at higher risk for increased aortic growth or aortic events (aortic dissection and/or prophylactic surgery) compared to MFS women [9]^-^[10]. Also, Tran-Fadulu and colleagues observed sex disparity in multigenerational families with TAA caused by mutations in the gene encoding the TGFβ receptor type 1 (*TGFBR1*) [34]–a finding that was recently confirmed in a larger international study [35]. In addition, there is a paucity of prospective data that assess the impact of pregnancy on aortic disease in women with heritable thoracic aortic disease. To date, pregnancy and aortic disease are still a widely debated issue. In contrast to the few prospective studies in selected women under strict follow-up that have been published, retrospective studies indicate an increased risk for aortic dissection during pregnancy [13–18]. Although this may be related to a publication bias towards the more severe cases, caution and extra surveillance of these women during pregnancy is strongly advised. In addition to the increased risk for dissection, there is concern that pregnancy triggers aortic growth and hence can induce an increased risk for aortic events in the long term [20]. Large, multicenter prospective studies are needed to clarify these issues. Our small retrospective study confirms the absence of aortic events but increased aortic growth rate during pregnancy in women with MFS. Of note, these women are under strict follow up in accordance with the European guidelines applicable during pregnancy.

Even fewer studies report the impact of sex and pregnancy on thoracic aortic disease in mouse models. This is a serious shortcoming, since sex-specific data in mice could potentially be of great value to gain insights into clinical management and treatment of patients. The only genetic TAA mouse model for which data on the impact of sex are available, is the *Alk5*^*iko*^ mouse. Selective deletion of *Alk5* or *Tgfbr1* in smooth muscle cells of adult mice results in thoracic aortic aneurysm and rupture in male mice, while female mice present only a subclinical phenotype with mild fragmentation of the aortic elastic lamellae [36]. In the current study, we provide evidence for a similar effect in GT-8/+ MFS mice. More severe aortic disease, manifesting as aneurysm and fragmentation of the elastic lamellae, is observed in male heterozygous GT-8/+ mice when compared to female mice. Strikingly, this difference is only observed when considering the nulliparous heterozygous GT-8/+ females. Multiparous heterozygous GT-8/+ female mice and male GT-8/+ mice have comparable aortic diameters and fragmentation of the aortic elastic lamellae. None of the mice suffered from aortic dissection or rupture. More multiparous GT-8/+ females did, however, present with aortic valve regurgitation compared to the male GT-8/+ mice.

The presence of several major breaks across the elastic lamellae per cross-section of the aortic root in male and multiparous female GT-8/+ mice suggests that severe fragmentation of the elastic lamellae is associated with dilatation of the thoracic aorta and that more severe fragmentation is associated with larger aortic diameters. In humans with aortic aneurysms requiring surgical replacement, pathological findings include severe fragmentation of the elastic lamellae and cystic medial degeneration. In addition, circulating fragments of fibrillin-1 may be biomarkers for aortic aneurysm and dissection [30]. Further study of the GT-8 Marfan mouse model will allow the identification of molecular and cellular mechanisms that cause initial fragmentation as well as major breaks in the elastic lamellae.

Interestingly, aortic dilatation in GT-8/+ Marfan mice was not restricted to the aortic root, but also included the ascending aorta and arch. This is in accordance with what has been observed in most other MFS mouse models, including *Fbn1*^*mgN/mgN*^, *Fbn1*^*mgR/mgR*^, and *Fbn1*^*C1039G/+*^ mice [24],[37],[38]. The involvement of a large part of the thoracic aorta in MFS mice is different from the common aortic phenotype seen in MFS patients, in whom the aortic dilatation is usually restricted to the level of the sinuses of Valsalva. Although far less common, the descending thoracic and abdominal aorta of MFS patients can also be affected. Schoenhoff and colleagues recently observed that the initial presentation in 8% of MFS patients was a descending or thoraco-abdominal aortic aneurysm [11]. To date, it is not clear why mice consistently develop aneurysms distal from the aortic root. Conceivably, hemodynamic differences between bipedal and quadrupedal mammals may influence this phenotypical difference.

Previously, it has been hypothesized that sexual dimorphism in aneurysm models is influenced by sex hormones. Several studies, however, documented contradictory results on the role of (exogenous and endogenous) female and male sex hormones on aneurysmal disease. Exposure of neonatal female mice to testosterone confers adult susceptibility to angiotensin II-induced ascending and abdominal aortic aneurysms [39]. Furthermore, ovariectomy in female *Alk5*^*iko*^ mice doubled the aortic disease penetrance, suggesting that in this model sexual dimorphism is in part the result of the protective effect of endogenous ovary-derived sex hormones [36]. The protective effect of 17β-estradiol has also been demonstrated in several elastase perfusion- and angiotensin-II infusion models of aortic aneurysms, as well as in genetic mouse models [40–45]. It has been suggested that estrogen exerts its effect by influencing serum lipid levels, inflammation and vessel wall homeostasis [42, 46, 47]. Previously, it has been shown that female sex steroids can modulate arterial structure and function, since 17β-estradiol and progesterone can decrease collagen deposition and increase elastin deposition in human aortic smooth muscle cell (HASMC) culture [48]. In addition, a combination of 17β-estradiol and progesterone, increased fibrillin-1 deposition in the matrix of cultured human aortic SMCs but only relative to testosterone, which significantly reduced fibrillin-1 expression compared to controls [48]. Since it is known that high levels of female sex hormones are associated with lower arterial stiffness, the authors suggested that female sex steroids influence this feature through modulation of extracellular matrix homeostasis [48].

In our study, we tested whether 17β-estradiol promotes fibrillin-1 production *in vitro*. Few compounds are known to increase fibrillin-1 synthesis and secretion. TGFβ was reported to increase fibrillin-1 matrix deposition without increasing fibrillin-1 protein or RNA expression [49]. We detected increased production of fibrillin-1 in response to TGFβ compared to controls. 17β-estradiol, added in physiological concentrations, proved to be more potent than TGFβ in the induction of fibrillin-1 by aortic smooth muscle cells. There are multiple possible explanations for the less severe aortic disease found in nulliparous Marfan mice, but our observation that 17β-estradiol increased the production of fibrillin-1 by smooth muscle cells raised the possibility that 17β-estradiol may improve aortic wall morphology and properties through this mechanism, which could in turn ameliorate aortic disease. If true, this would open the door to approaches aimed at increasing fibrillin-1 synthesis as possible treatments for aortic aneurysm. Future research is definitely warranted.

We expect that, if protection of females is due to 17β-estradiol stimulation of fibrillin-1 production, *Fbn1*^*C1039G/+*^ mice will show the same sex differences as GT-8/+ mice. In contrast, we would predict that *Fbn1*^*mgR/mgR*^ mice would not display sex differences in aortic disease, since in this model *Fbn1* expression is artificially repressed by the presence of the Neomycin cassette (to 20% of wild-type) [27]. Thus, any factor increasing the expression or biosynthesis of fibrillin-1 would not be expected to show an effect in *Fbn1*^*mgR/mgR*^ mice. This hypothesis should be tested in future studies.

Despite the possibility that 17β-estradiol has a protective effect in female MFS mice, pregnant MFS mice showed more severe aortic disease than nulliparous female mice. Moreover, the aortic phenotype of the multiparous GT-8/+ females was comparable to male MFS mice and sometimes even worse. This may seem contradictory, since there is an increase in female sex hormones, such as 17β-estradiol and progesterone, during pregnancy. Aortic root growth (1.2 mm on average) occurs in healthy women during pregnancy, and the aortic root remains enlarged for up to six weeks after delivery [33]. It is assumed that while these effects can be largely disregarded in healthy women their impact on an underlying diseased aorta may have severe consequences, as is reflected for example in an adverse immediate and long-term aortic outcome in patients with Marfan syndrome [20, 21]. Pregnancy also affects the aorta in wild-type mice to a minor extent. Multiparous wild-type females have slightly larger mean aortic diameters than nulliparous wild-type females, but no significant differences are observed between both groups at 8 months of age (no statistical analyses performed at 12 month time point). This result supports the abovementioned assumption that an underlying aortopathy is required for pregnancy to have a marked adverse effect on the aorta.

Multiple factors impact the aorta during pregnancy. In addition to the hormonal changes inherent to pregnancy, which are meant to soften tissues in preparation for delivery and increase tissue fragility, hemodynamic changes also occur. Increased cardiac output required for adequate placental perfusion will result in increased wall shear stress in the aorta [50, 51]. Also, increased outflow resistance is present in the arterial tree in later stages of pregnancy, due to compression of the abdominal aorta and iliac arteries by the uterus [50]. Possibly, these hemodynamic effects undo the female sex hormone-related protective effect and result in increased aneurysm formation. Indeed, in the *Alk5*^*iko*^ mouse model it was shown that norepinephrine-induced hypertension breaks the female sex protective effect [50]. Future studies in our MFS mouse model are warranted in order to unravel underlying disease mechanisms and determine the influence of different factors, such as hormones and hemodynamics on these mechanisms.

In conclusion, this is the first study providing evidence for an effect of sex and pregnancy on both the dimensions and morphological characteristics of the aorta in MFS mice. In addition, we showed that 17β-estradiol promotes fibrillin-1 production in human aortic smooth muscle cells and suggest that compounds that increase fibrillin-1 production in a Marfan diseased aorta may have therapeutic potential for aortic aneurysms.

## Supporting information

S1 Table Aortic diameters in wild-type and GT-8/+ mice at 8 and 12 months of age. Measurements are shown as mean ± standard deviation. P-values of the one-way ANOVA analyses are listed. * The 12 months old wild-type multiparous female mice were excluded from the ANOVA analyses because n was too low.
(XLSX)

S2 Table Progressive aortic diameters in male wild-type and GT-8/+ mice. A detailed cardiovascular ultrasound study at 1, 3, 6, 8 and 12 months in mutant and wild-type male mice showed that male heterozygous GT8/+ mice are characterized by more extensive progressive dilatation of the thoracic aorta compared to wild-type littermates. Although there was a trend towards larger aortic diameters in GT8/+ mice as early as 1 month of age, aortic diameters were only significantly increased compared to wild-type mice at later time points. The aortic root diameter of GT-8/+ males is significantly increased compared to wild-types males from the age of 3 months. The proximal ascending aorta diameter is significantly larger only in 12 months old GT-8/+ males compared to wild-types. Both the distal ascending aorta and arch show significant differences in diameter from the age of 6 months. Results are given as mean ± standard deviation. P-values were calculated using an unpaired student T-test. AR: aortic root; PAscAo: proximal ascending aorta; DAscAo: distal ascending aorta; DescAo: descending aorta.
(XLSX)

S1 Fig Aortic root morphology of 8 months old wild-type and heterozygous GT-8/+ males and females. Original full cross section micrographs of the images used in Fig 3A.
(TIF)

S2 Fig Graphs of fragmentation of the aortic elastic lamellae in mice between 12–16 months of age. Increased numbers of major breaks in aortic elastic lamellae were seen in GT-8/+ males and females compared to the wild-type controls (ANOVA p = 0.0002). The numbers of major breaks in multiparous GT-8/+ females is higher than in nulliparous GT-8/+ females and GT-8/+ males. Each symbol represents a single animal. Means with standard deviation are shown; p-values: * <0.05, ** <0.005, *** <0.001. F: female; M: male; np: nulliparous; mp: multiparous.
(TIF)
