# Correction: Sex, pregnancy and aortic disease in Marfan syndrome

**DOI:** 10.1371/journal.pone.0197631

**Published:** 2018-05-14

**Authors:** Marjolijn Renard, Laura Muiño-Mosquera, Elise C. Manalo, Sara Tufa, Eric J. Carlson, Douglas R. Keene, Julie De Backer, Lynn Y. Sakai

In Table 1, the z-scores for aortic root diameter at baseline were calculated based on an older version of the formula (Roman et al. 1989) instead of the more recent and generally accepted updated formula (Devereux et al. 2012). Please see the corrected Table 1 here.

**Table 1:** Characteristics of the aortic root of MFS patients at baseline and after several years of follow-up.

	men	women		
		non-pregnant	pregnant	p-value
n	10	10	10	
age (year)	26 (±10)	27 (±6)	27 (±3)	0.899
aortic root at baseline (mm)	40.8 (±3.52)	36.9 (±2.97)	35 (±3.40)	**0.002**^a^
Z-scores at baseline	2.67 (±1.23)	2.87 (±1.24)	1.76 (±1.40)	0.142
aortic root at follow-up (mm)	42.5 (±2.80)	37.9 (±3.10)	38.7 (±3.52)	**0.007**^b^
follow-up time (year)	5.00 (±2.75)	5.09 (±2.66)	6.63 (±3.74)	0.341
aortic root growth rate (mm/year)	0.36 (±0.35)	0,.2 (±0.39)	0.64 (±0.42)	**0.022**^c^

Results are given as means with the standard deviation between brackets. Significant p-values (<0.05) are indicated in bold.

^a^ Values are significantly different between men and non-pregnant and pregnant women (p = 0.040 and p = 0.002, respectively), but not between the non-pregnant and pregnant women (p = 0.623).

^b^ Values are also significantly different when pairwise comparing men and non-pregnant and pregnant women (p = 0.010 and p = 0.038, respectively).

^c^ Significant difference only between non-pregnant and pregnant women (p = 0.018). No significant difference was observed between men and non-pregnant (p = 0.559) or pregnant women (p = 0.352).
