# Supplementary figures and images for: Sex, pregnancy and aortic disease in Marfan syndrome
Source: PLoS One. 2017 Jul 14;12(7):e0181166. doi: 10.1371/journal.pone.0181166 (PMC5510874; doi:10.1371/journal.pone.0181166)

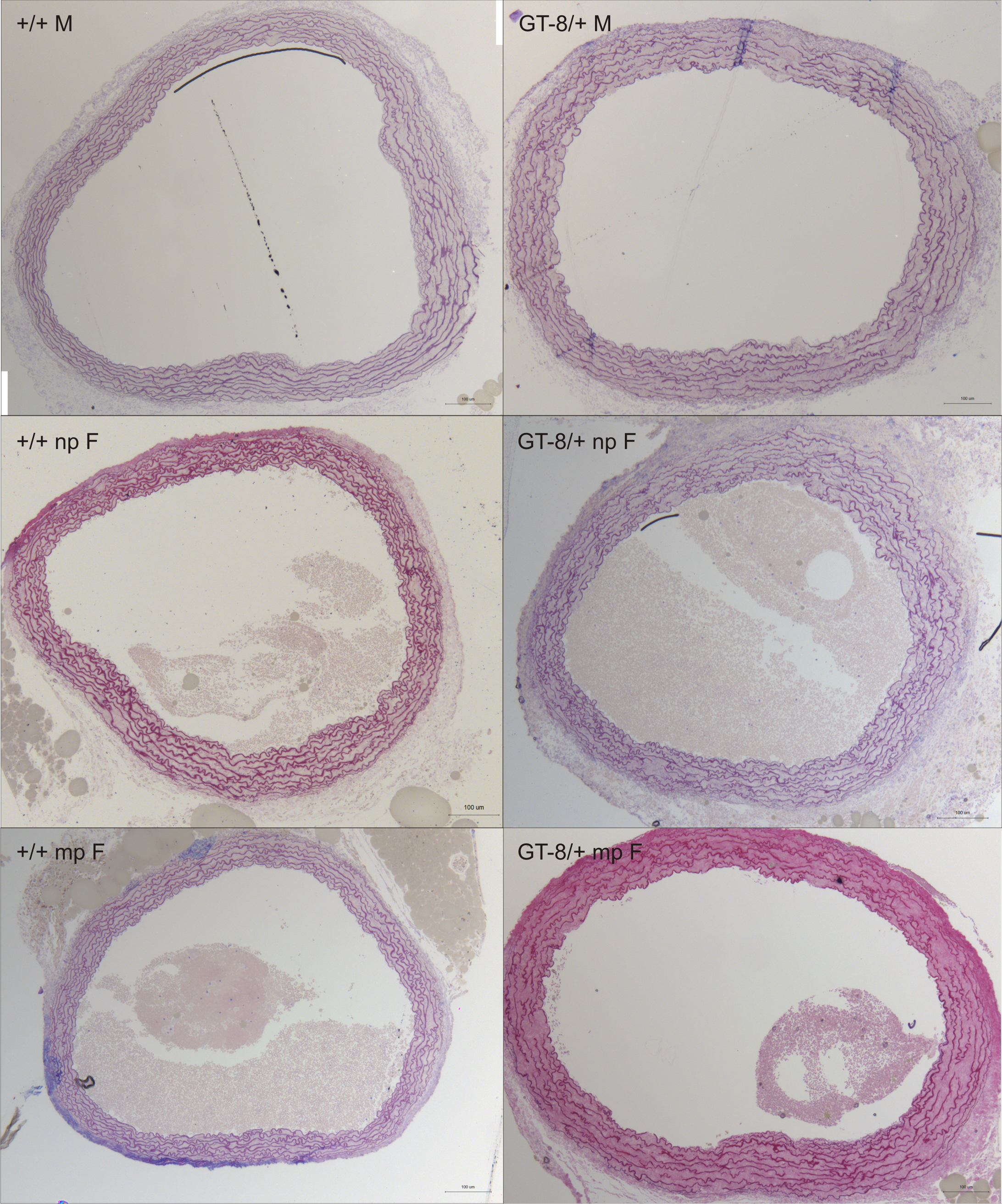

Supplement: S1 Fig — Original full cross section micrographs of the images used in Fig 3A. (TIF) [file pone.0181166.s003.tif]

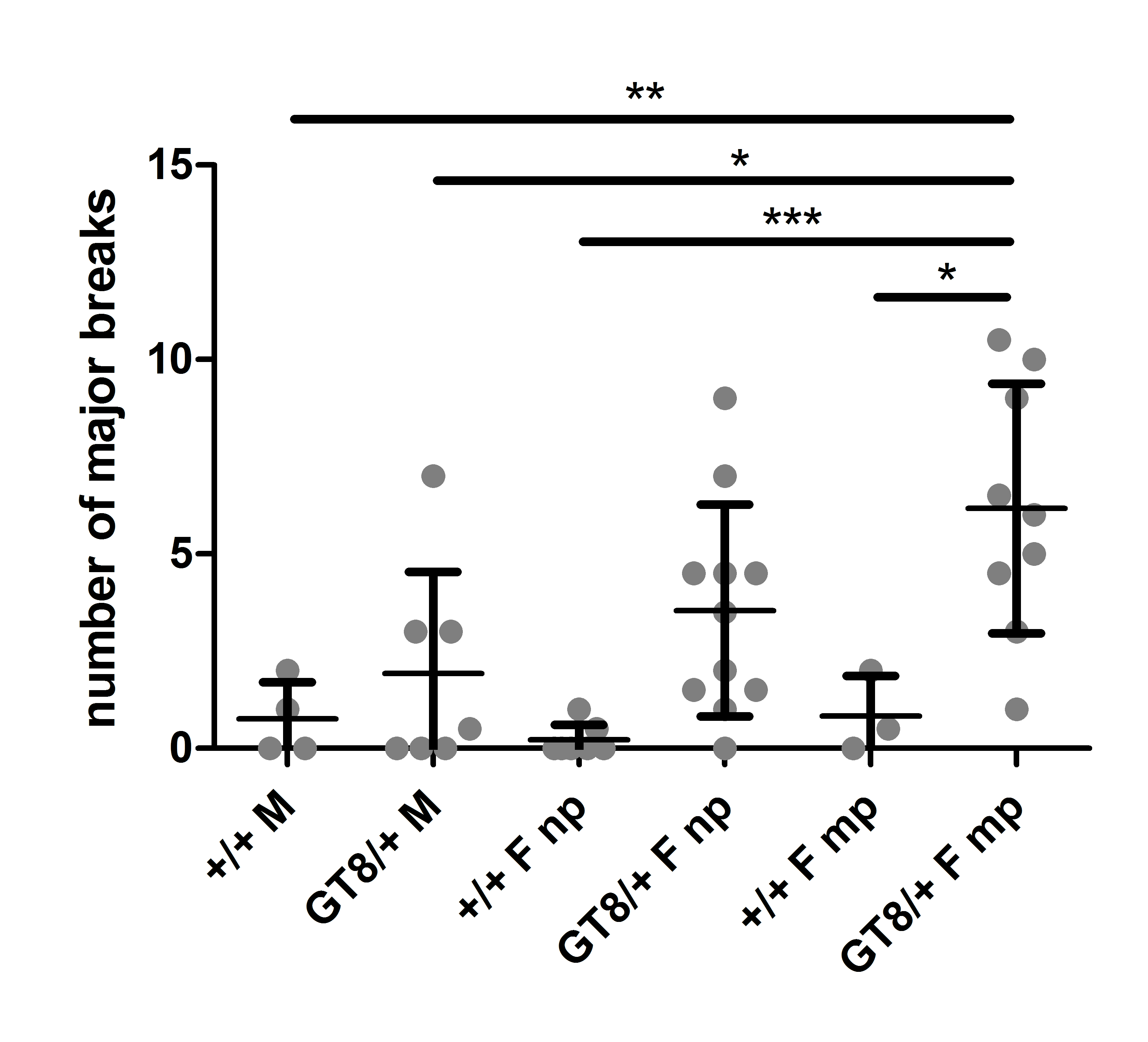

Supplement: S2 Fig — Increased numbers of major breaks in aortic elastic lamellae were seen in GT-8/+ males and females compared to the wild-type controls (ANOVA p = 0.0002). The numbers of major breaks in multiparous GT-8/+ females is higher than in nulliparous GT-8/+ females and GT-8/+ males. Each symbol represents a single animal. Means with standard deviation are shown; p-values: * <0.05, ** <0.005, *** <0.001. F: female; M: male; np: nulliparous; mp: multiparous. (TIF) [file pone.0181166.s004.tif]
